# Checked Perspiration

**Published:** 1884-02

**Authors:** 


### CHECKED PERSPIRATION

Is the fruitful cause of sickness, disease and death to multitudes every year. If a tea-kettle of water is boiling on the fire, the steam is seen issuing from the spout, carrying the extra heat away with it, but if the lid be fastened down and the spout be plugged, a destructive explosion follows in a very short time.

Heat is constantly generated within the human body, by the chemical disorganization, the combustion, of the food we eat. There are seven millions of tubes or pores on the surface of the body, which in health are constantly open, conveying from the system by what is called insensible perspiration this internal heat, which having answered its purpose, is passed off like the jets of steam which are thrown from the escape-pipe, in puffs, of any ordinary steam engine ; but this insensible perspiration carries with it, in a dissolved form, very much of the waste matter of the system, to the extent of a pound or two or more every twenty-four hours. It must be apparent, then, that if the pores of the skin are closed. if the multitude of valves, which are placed over the whole surface of the human body are shut down, two things take place. First, the internal heat is prevented from passing off, it accumulates every moment, the person expresses himself as burning up, and large draughts of water are swallowed to quench the internal fire—this we call "*Fever.*" When the warm steam is constantly escaping from the body in health, it keeps the skin moist, and there is a soft, pleasant feel and warmth about it. But when the pores are closed, the skin feels harsh, and hot, and dry.

